# A comparative study of the frequency of myofibroblasts and macrophages between the oral and cutaneous squamous cell carcinoma

**DOI:** 10.15171/joddd.2019.039

**Published:** 2019

**Authors:** Samira Mostafazadeh, Paria Emamverdizadeh, Khadijeh Abdal, Sevda Sadat Forghani

**Affiliations:** ^1^Department of Oral and Maxillofacial Pathology, Faculty of Dentistry, Urmia University of Medical Sciences, Urmia, Iran; ^2^Department of Oral and Maxillofacial Pathology, Faculty of Dentistry,Tabriz University of Medical Sciences, Tabriz, Iran; ^3^Department of Oral Pathology, Faculty of Dentistry, Ilam University of Medical Sciences, Ilam , Iran; ^4^Privet Practice, Urmia, Iran

**Keywords:** Cutaneous squamous cell carcinoma, macrophage, myofibroblast, oral squamous cell carcinoma

## Abstract

***Background.*** Oral squamous cell carcinoma (OSCC) is among the ten most frequent malignant tumors, with SCC accounting for 94% of oral malignancies. Myofibroblasts and macrophages are multifunctional cells that have a crucial role in the biological behavior of these tumors. This study aimed to comparatively evaluate the frequency of myofibroblasts and macrophages between oral and cutaneous squamous cell carcinomas.

***Methods.*** Sixty paraffin blocks, consisting of 20 cases of OSCC, 20 cases of CSCC, 10 cases of normal skin, and 10 cases of normal oral mucosa, were selected for this descriptive-analytical cross-sectional study. To evaluate the prevalence of myofibroblasts, α-SMA staining and CD163 markers for macrophages were used. In this study, the data were analyzed with Wilk-Shapiro test and t-test using SPSS 19. Statistical significance was set at P<0.05.

***Results.*** The mean myofibroblast scores in CSCC and OSCC were 20.05 and 20.95, respectively, with no significant difference between the means (P>0.05). The mean macrophage scores in the skin and oral cavity were 28.125 and 49.67, respectively, with a statistically significant difference (P<0.05), indicating that the mean oral macrophage score was significantly higher than that in the skin. There was no significant difference between the presence and accumulation of macrophages and myofibroblasts between the oral and cutaneous SCCs; however, the intensity of accumulation and color pattern in OSCC and CSCC were higher than those in the normal skin and mucosa (P<0.05).

***Conclusion.*** According to the results of this study, it appears the biological behavior of OSCC and CSCC does not depend on myofibroblasts, and other factors might be involved.

## Introduction

Cancer is the second most important cause of death in developed countries and the third cause of death in developing countries after cardiovascular diseases.^1^ Five percent of all tumors occur in the head and neck region, with half of them in the oral cavity.^2^ Cancers of this area are one of the major reasons for mortality and morbidity worldwide and reported as the most common cancers in humans.^3,4^ Oral squamous cell carcinoma (OSCC) is the most common oral cancer, and cutaneous squamous cell carcinoma (CSCC) is a frequent cancer of the skin.^5^ Skin malignancy, which has been on the increase recently, accounts for 11% of all human malignancies. SCC accounts for approximately 20% of skin tumors and originates from dysplastic surface epithelium.^6,7^ Ability to predict the biological behavior of oral and cutaneous SCC helps prepare an appropriate treatment plan.^8^ In addition to the early diagnosis of the condition, identification of tumor growth and development inhibitors is effective in increasing patient survival rate and improving the quality of life.^9^ In solid tumors, including SCC, a combined effect of cancer and stromal cells (i.e., fibroblasts and endothelial and inflammatory cells) plays a pivotal role in tumor progression, angiogenesis, local invasion, recurrence, and metastasis.^10,11^

Myofibroblasts have a critical role in regulating the stroma under normal and pathological conditions via direct cell-to-cell contacts and by secreting matrix metalloproteinases (MMPs), MMP tissue inhibitors, extracellular growth factors, cytokines, chemokines, and fatty products by expressing specific receptors. Apart from their role in wound healing processes, Myofibroblasts are indispensable for the induction of tissue and mucosal immunity and stem cell donation.^12^ Carcinoma-related fibroblasts, including myofibroblasts, are often seen in the stroma of human carcinoma. The differentiation of fibroblasts into myofibroblasts due to the effect of cytokines and growth factors originating from tumor cells is a principal and crucial event in tumorigenesis. The induction of myofibroblasts is due to the factors caused by OSCC that stimulate carcinoma and neoplastic growth.^13^ The high stromal myofibroblast counts are associated with a poor prognosis of the oral, breast, and colorectal carcinomas.^14^ Macrophages have a pivotal role in the defense mechanisms of the host from various perspectives and also in pathophysiologic conditions, including chronic inflammatory diseases and cancer. A plethora of studies have indicated that macrophages might inhibit or stimulate tumor growth. Tumor-associated macrophages (TAM) secrete proteome factors, including MMP9 and MMP11.^15^ The critical role of tumor-associated macrophages was initially clarified in primary tumors; however, subsequent studies showed that these macrophages contributed to carcinogenesis and indicated a relationship between high counts of TAMs and poor prognosis in certain cancers.^16^ TAMs can lead to a lack of diagnosis of tumor antigens and can release factors that induce tumor growth and angiogenesis.

Furthermore, SCC can provide an environment that converts macrophages into TAMs and plays a role in tumor growth.^17^ An increase in TAM counts can correlate with the duration of OSCC progression, resulting in angiogenesis and an increase in malignancy.^15^ On the other hand, the prognosis and the potential for malignancy and metastases of OSCC and CSCC are different. For OSCC, the 5-year survival rate is 35–45%, and the chance of metastasis is 40–50%.^17^ Concerning CSCC, in the head and neck area, the 5-year survival rate is 54%, and the chance of metastasis is 11.7%.^18^ This study aimed to compare myofibroblast and macrophage counts between OSCC and CSCC to determine whether myofibroblasts and macrophages can contribute to a better understanding of the differences in the biological behaviors of these neoplasms.

## Methods

This descriptive analytical/cross-sectional study was carried out after approval and obtaining the necessary permissions from the Ethics Committee of the Faculty of Dentistry, Urmia University of Medical Sciences. In this study, paraffin blocks of patients referred to Imam Khomeini Hospital of Urmia, with oral and skin squamous cell carcinoma, were selected.

The sample size in the study was estimated at 60 paraffin blocks, comprising 20 cases of OSCC, 20 cases of CSCC, 10 cases of normal skin and 10 cases of normal oral mucosa, which were assigned to 4 groups:

**Group 1:** 10 blocks of normal oral mucosa

**Group 2:** 10 blocks of normal skin

**Group 3:** 20 blocks of oral squamous cell carcinoma

**Group 4:** 20 blocks of squamous cell carcinoma of the skin

Forty SCC blocks were selected, 20 of which belonged to male, and 20 belonged to female subjects, with an age range of 42–81 years.

All the necessary information, including the location of the lesion and the pathologic diagnosis of the lesion, were obtained from the patient files. Incomplete files, in terms of patient data, lack of a definite diagnosis of the lesion, and inadequacy of the block samples for staining procedures, were excluded from the study. Patients included in the study had no history of any disease, such as allergy, infection, and previous tumors. To determine the tumor grade, 5-µm sections were prepared from the paraffin blocks and stained with hematoxylin and eosin technique (H&E). Then the samples were evaluated by two pathologists under a light microscope based on the reference book criteria. Grade 1 oral and skin squamous cell carcinoma samples were selected.

Immunohistochemistry staining (IHC) was applied to determine myofibroblast and macrophage counts.

α-SMA staining and CD163 markers were used to determine the frequency of myofibroblasts and macrophages, respectively; after preparing the microscopic slides, the samples were evaluated by oral and general pathologists under a light microscope (Olympus Ch30, Japan). In this study, the data were analyzed with the Wilk-Shapiro test and t-test using SPSS 19. Statistical significance was set at P<0.05. Informed consent forms were signed by all the patients before evaluating the samples.

## Results

**Figure 1:**
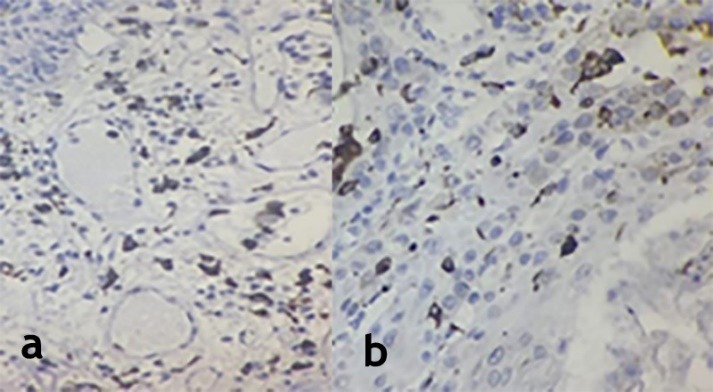
CD163-positive macrophages in OSCC, ×100 (a), and CSCC, ×100 (b) magnifications.

**Figure 2:**
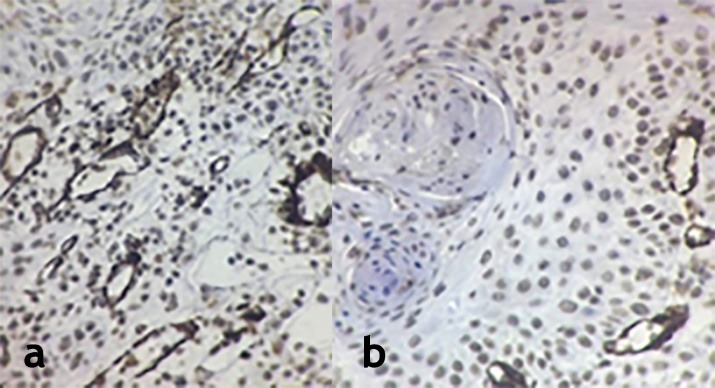
SMA-positive myofibroblasts in CSCC at ×100 (a), and OSCC at ×100 (b) magnifications.

Sixty samples, 20 OSCC, 20 CSCC, 10 normal oral mucosa (NM) and 10 normal skin (NS) cases, were evaluated. Differences in the staining intensity of macrophages and myofibroblasts in the OSCC and CSCC samples were evident (Figures 1and 2).

Of the 20 samples tested to determine the frequency of myofibroblasts in the OSCC samples, the α-SMA staining was poor in 13 cases (1‒25%), medium in four (25–50%), and high in three (50‒75%) (Table 1).

**Table 1:** The density of myofibroblasts in all the samples

**Group**	**Negative**	**Poor**	**Moderate**	**Severe**
**Normal skin**	6 (60%)	4 (40%)	0 (0%)	0 (0%)
**Normal oral mucosa**	6 (60%)	4 (40%)	0 (0%)	0 (0%)
**cutaneous squamous cell carcinoma**	0 (0%)	13 (65%)	6 (30%)	1 (5%)
**oral squamous cell carcinoma**	0 (0%)	13 (65%)	4 (20%)	3 (15%)

In determining the frequency of macrophages in the OSCC samples, a total of 20 samples were stained; CD163 staining was poor in three cases (1–25%), medium in seven (25‒50%), high in eight (50–75%), and very high in two (75‒100%) (Table 2).

**Table 2:** The density of macrophages in all the samples

**Group**	**Negative**	**Poor**	**Moderate**	**Severe**
**Normal skin**	6 (60%)	4 (40%)	0 (0%)	0 (0%)
**Normal oral mucosa**	6 (60%)	4 (40%)	0 (0%)	0 (0%)
**cutaneous squamous cell carcinoma**	0 (0%)	11 (55%)	7 (35%)	2 (10%)
**oral squamous cell carcinoma**	0 (0%)	3 (15%)	7 (35%)	10 (50%)

Of the 20 samples tested to determine the frequency of myofibroblasts in the CSCC samples, the α-SMA staining was poor in 13 cases (1‒25%), medium in six (25‒50%), and high in one (50‒75%).

Of the 20 samples tested to determine the frequency of macrophages in the CSCC samples, CD163 staining was poor in 11 cases (1‒25%), medium in seven (25‒50%), and high in two (50–75%).

The myofibroblast mean percentage in the CSCC samples was 20.05%, while it was 20.95% in the OSCC samples (P>0.05), with no significant difference between the myofibroblast percentages between the OSCC and CSCC samples.

The macrophage mean percentage was 28.125% in the CSCC and 49.67% in the OSCC samples, with a difference of 21.54% (P<0.05), which was significant statistically. The mean percentage of OSCC macrophages was higher than that of CSCC (Table 3). There was no significant difference between the presence and accumulation of macrophages and myofibroblasts between CSCC and normal mucosa, but the expression and density of macrophages in OSCC and CSCC were higher than those in the normal skin and mucosa (P<0.05).

**Table 3:** The mean percentages of myofibroblasts and macrophages in all the samples

**Group**	**Mean of myofibroblast** **density**	**Mean of macrophage** **density**	**Std. Deviation** **macrophage**	**P-value** **macrophage**	**Std. Deviation** **myofibroblast**	**P-value** **myofibroblast**
**Cutaneous squamous cell carcinoma**	1.49	28.125	15.00652	0.149	37842.11	0.007
**Oral squamous cell carcinoma**	395.60	49.67	20.40274	0.337	55499.14	0.003

## Discussion

It seems that the frequency of myofibroblasts and macrophages between the OSCC and CSCC might explain the biological behaviors of these tumors. The findings of this study indicate that macrophages in the OSCC appear at a moderate and intense level during staining, while most myofibroblasts appear at a weak level. This difference in staining was also reported in a study by Sapp et al^19^ in 1997.It seems that the presence and accumulation of macrophages against myofibroblasts in OSCC play an important role in determining tumor progression and also as a factor for predicting prognosis and therapeutic goals, which is an indication for further studies in this field. The findings of the current study suggest that the presence and accumulation of myofibroblasts do not help distinguish tumor progression in CSCC. This finding was consistent with those of the studies by Vered et al (2009) and Saif et al (2010).^20,21^ Dodani et al^22^ (2014) showed no significant differences in the presence and accumulation of myofibroblasts in OSCC and CSCC in terms of the intensity and pattern of staining,^22^ consistent with the results of the present study. This finding in our study contradicts the findings of a study by de-Assis et al^23^ in 2012, who reported a statistically significant difference in the presence and accumulation of myofibroblasts in OSCC and normal mucosa.^23^

Comparison of the frequency of macrophages between OSCC and CSCC showed that the macrophage mean percentage in OSCC was significantly higher than that in CSCC. In this regard, no similar or contradictory findings were found in the literature review carried out by the researchers.

Other findings of this study indicated no significant difference in the frequencies of myofibroblasts between the skin and normal mucosa and between OSCC and CSCC. In the current study, the density and staining intensity of macrophages in OSCC and CSCC were higher than skin and normal mucosa, which might be important in the progression of inflammation and SCC. Due to the novelty of this issue and a lack of similar studies on the differences between the types of inflammatory cells in SCC types, it is recommended that such a relationship be examined in future studies.

It is recommended that the data available in the Cancer Registration Center be used in further studies, including factors such as smoking and alcohol in more numerous groups, and continuing education programs be planned to increase the awareness of dentists about the early diagnosis and appropriate treatment for SCC.

## Conclusion

Considering the findings of this study, it does not seem that the biologic behavior of OSCC and CSCC depends on myofibroblasts, and other factors might have a role. There might be a relationship between the presence and accumulation of macrophages in comparison with myofibroblasts in the OSCC in terms of the severity and pattern of staining, which might play a crucial role in diagnosing tumor progression as well as a factor for the prediction of prognosis and therapeutic goals. However, confirmation of this finding requires further studies in this regard.

## Acknowledgments

This paper was extracted from a doctoral thesis and financially supported by the Research Council of Urmia University of Medical Sciences.

## Authors’ contributions

SM and KA were responsible for the concept and the design of the study. SM and SSF performed the data collection and statistical analysis. KA and PE were responsible for revision of manuscript and final approval to be published.

## Competing Interests

The authors declare that they have no conflict of interests.

## Funding

This paper was extracted from the thesis and financially supported by the Research Council of Urmia University of Medical Sciences

## Ethics approval

This research was approved by the Ethics Committee of Urmia University of Medical Sciences. All the patient data were confidential and written informed consent was obtained from all the patients before being included in the study.
